# Prognostic stratification of oropharyngeal cancer patients in a betel nut chewing and low HPV area

**DOI:** 10.1186/s40463-023-00632-x

**Published:** 2023-04-20

**Authors:** Huai-Pao Lee, Ching-Chih Lee

**Affiliations:** 1 Department of Pathology and Laboratory Medicine, Kaohsiung Veterans General Hospital, Kaohsiung, Taiwan; 2 Department of Nursing, Meiho University, Pingtung, Taiwan; 3 Department of Otolaryngology, Head and Neck Surgery, Kaohsiung Veterans General Hospital, No.386, Dazhong 1St Rd., Zuoying Dist., Kaohsiung City, 81362 Taiwan (R.O.C.); 4 School of Medicine, National Defense Medical Center, Taipei, Taiwan; 5 Department of Otolaryngology, Head and Neck Surgery, Tri-Service General Hospital, Taipei, Taiwan; 6 Institute of Hospital and Health Care Administration, National Yang Ming Chiao Tung University, Taipei, Taiwan

**Keywords:** Oropharyngeal cancer, p16, Epidermal growth factor receptor (EGFR), Survival, Prediction

## Abstract

**Background:**

This study aimed to establish a simple predictive model for oropharyngeal cancer (OPC) in an area with a relatively low prevalence of human papillomavirus (HPV) and frequent betel nut chewing.

**Methods:**

A total of 116 patients with OPC were recruited from the clinical research database of a referral cancer center between 2013 and 2018. Patient characteristics—including age, gender, tumor stage, differentiation, and treatment modality—were extracted from the database. Patients diagnosed after 2018 were staged using the 7th AJCC staging system to explore the impact of extra-nodal tumor extension (ENE) on survival. Immunohistochemical analysis was performed for p16, epidermal growth factor receptor (EGFR), p53, and programmed cell death ligand 1 (PD-L1). ENE status was evaluated by pathological analysis or radiological features. Primary outcome was disease-specific overall survival (OS). Univariate and multivariate Cox regression were used to establish a predictive model.

**Results:**

Mean age was 57.3 ± 9.9 years; 103 patients (88.8%) were male. P16 positive OPC was positively associated with higher PD-L1 and a tonsillar sub-site and negatively associated with betel nut chewing and cigarette smoking. In Cox regression, age, p16 status, EGFR, cT4, ENE, and cigarette smoking were significantly associated with OS. In survival tree analysis, cT stage was the most important risk stratification parameter, followed by EGFR expression and p16 status. Patients with cT4 stage or high EGFR were classified as the high-risk group and had poorest OS.

**Conclusions:**

Due to the low prevalence of HPV and popularity of betel nut chewing in Asia, the relative importance of prognostic predictors for OPC are not identical to Western countries. Identification of significant prognostic biomarkers may improve treatment.

*Trial registration* This study was registered and approved by the Institutional Review Board (IRB) of Kaohsiung Veterans General Hospital (VGHKS19-CT9-07; date of approval: Aug 9, 2019).

**Graphic Abstract:**

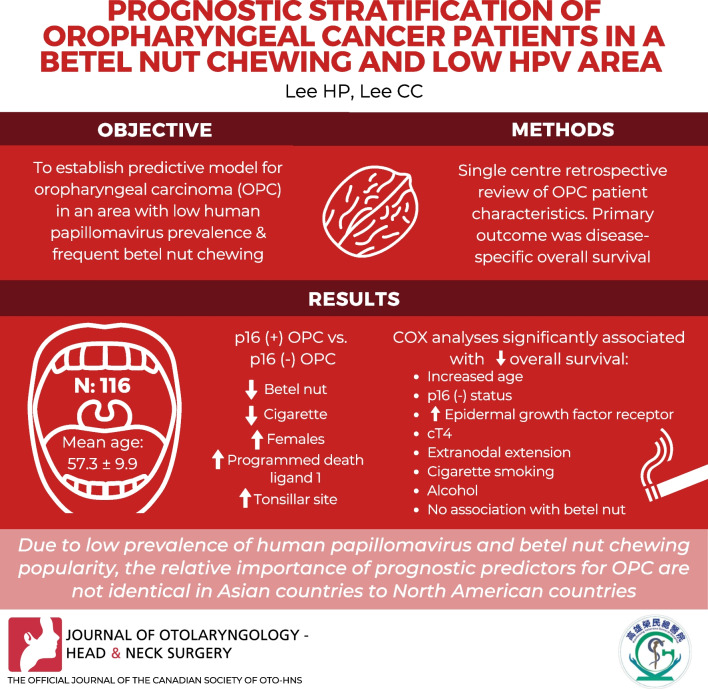

**Supplementary Information:**

The online version contains supplementary material available at 10.1186/s40463-023-00632-x.

## Introduction

Among the biomarkers for head and neck cancer, p16 status has been demonstrated to have prognostic significance in oropharyngeal cancer (OPC); p16-positive OPC is associated with better outcomes than p16-negative OPC [1–3]. P16 immunohistochemical staining has also been used as a surrogate marker of human papillomavirus (HPV)-related OPC [1, 4]. The different outcomes of HPV-related OPC and non-HPV-related OPC have been integrated into two staging systems in the AJCC 8th edition [5].

In addition to p16 status, several biomarkers such as epidermal growth factor receptor (EGFR), programmed cell death ligand 1 (PD-L1), and p53 have been investigated in OPC. Overexpression of EGFR has been reported to have negative prognostic impact in OPC [6, 7]. P16-negative and EGFR-positive OPC have significantly poorer outcomes than p16-positive and EGFR-negative OPC [6].

PD-L1 expression in head and neck cancer cells and tumor-associated immune cells is evaluated in order to predict the response to anti-programmed death 1 (PD-1) receptor antibody treatment, such as pembrolizumab [8]. High PD-L1 expression is associated with better immunotherapeutic benefits in head and neck cancer, irrespective of HPV status [8]. Although high PD-L1 expression is associated with a better prognosis after immunotherapy in HPV-related OPC than other subtypes of OPC [9], the prognostic impact of PD-L1 expression regardless of immunotherapy remains to be elucidated.

Mutation of the tumor suppressor gene *TP53* can be detected by p53 immunohistochemical staining and is prevalent in head and neck cancers associated with non-HPV carcinogens such as tobacco or alcohol [10]. The p16-positive and p53 wild-type immunophenotypes of OPC are reported to have better survival rates than p16-negative and mutant p53 OPC [11]. In addition to these biomarkers, several clinical prognostic factors such as extra-nodal tumor extension (ENE) and T stage have major impacts on outcomes in OPC. Indeed, ENE has been incorporated into the AJCC 8th edition of the head and neck cancer staging systems and the presence of ENE upstages nodal status [5].

However, few studies have examined risk stratification of OPC in regions with a low incidence of HPV-positive OPC or in areas where betel nut chewing is popular. Thus, the aim of this study was to establish a simple predictive model for the prognosis of OPC based on biomarkers and clinical parameters in a region with a relatively low prevalence of HPV and where betel nut is commonly chewed.

## Methods

### Ethics statement

This study was approved by the Institutional Review Board (IRB) of Kaohsiung Veterans General Hospital (VGHKS19-CT9-07; date of approval: Aug 9, 2019). The requirement for written informed consent was waived because all personal identifying information had been removed from the dataset prior to analysis. This study followed the Strengthening the Reporting of Observational Studies in Epidemiology (STROBE) statement: guidelines for reporting observational studies [12].

### Patient recruitment and data collection

Using the clinical research database of Kaohsiung Veterans General Hospital, 139 patients diagnosed with OPC between 2013 and 2018 were initially recruited to this study. Patients with unknown survival status, unknown tumor differentiation, unknown treatment modality, unknown T stage, unknown N stage, unknown alcohol or betel nut consumption status, or unknown smoking status were excluded; 116 patients were finally included. Patient characteristics, such as age, gender, tumor stage, differentiation, and treatment modality, were extracted from the database. The choice of treatment was based on the National Comprehensive Cancer Network (NCCN) guidelines, patients’ choices and physicians’ suggestions. Although induction chemotherapy (IC) was regarded as category 3 for patients with cT3-4 or cN2-3 disease, IC might reduce recurrence in patients followed by chemoradiotherapy, and preserve function in patients with surgery [13]. Our data (not shown) also showed a positive association between IC and the advanced stage. Patients diagnosed after 2018 were staged using the 7th AJCC staging system to explore the impact of extra-nodal extension (ENE) on outcomes. ENE status was evaluated according to the radiological features or pathological findings if patients underwent surgery. The main outcome of this study was disease-specific overall survival (OS).

### Tissue microarray

We selected representative paraffin-embedded oropharyngeal squamous cell carcinoma samples for the 116 patients and constructed tissue microarrays (TMA).

### Immunohistochemistry

Immunohistochemical (IHC) analysis of the tumor specimens for p16, EGFR, p53, and PD-L1 was performed using the TMA sections. P16, EGFR, and p53 staining was performed using standard reagents and techniques on a Bond III Automated Staining System (Leica Biosystems, Wetzlar, Germany). The sections were incubated with primary antibodies followed by the Bond Polymer Refine detection system (DS9800, Leica Biosystems, Newcastle upon Tyne, UK). The primary antibodies were P16 (clone JC2, 1:100; Zytomed Systems GmbH, Berlin, Germany), EGFR (clone EP22, 1:50; Zeta Corporation, Sierra Madre, CA, USA), and p53 (clone DO7, 1:200; Leica Biosystems). IHC for PD-L1 was performed using the PD-L1 clone 22C3 pharmDx kit on a Dako Autostainer Link 48 platform (clone 22C3, 1:50; Dako, Carpenteria, CA, USA). Positive and negative controls were prepared according to the manufacturers’ instructions.

A senior pathologist reviewed and scored all slides. P16 positivity was defined as diffuse, strong nuclear and cytoplasmic staining in ≥ 70% tumor cells (Additional file 1: Fig. S1A; Additional file 2: Fig. S1B) [14]. Strong nuclear p53 staining in ≥ 80% tumor cells was recorded as p53 mutant-type (Additional file 3: Fig. S2A; Additional file 4: Fig. S2B) [10]. The percentages of tumor cells with membranous EGFR staining were multiplied by the staining intensity score (1 + : weak; 2 + : intermediate; and 3 + : strong) to obtain the H-score for EGFR immunoreactivity, which ranges from 0 to 300 [6, 7]. A H-score ≥ 200 was considered as high EGFR expression (Additional file 5: Fig. S3A; Additional file 6: Fig. S3B). PD-L1 expression was assessed using the combined positive score (CPS), which is defined as the number of PD-L1 positive tumor and mononuclear inflammatory cells at any intensity within the tumor and adjacent tumor stromal area divided by the total number of viable tumor cells, multiplied by 100 [8]. A CPS ≥ 20 was recorded as high PD-L1 expression (Additional file 7: Fig. S4A; Additional file 8: Fig. S4B).

### Statistical analysis

All analyses were performed using SPSS statistical software (version 20, IBM Corporation, Armonk, NY, USA). Continuous variables were analyzed with one-way ANOVA and categorical variables were compared with Pearson’s Chi-square test or Fisher’s exact test. Kaplan–Meier survival curves were constructed for different groups and compared with the log-rank test. Univariate Cox regression analysis was used to identify factors significantly associated with 5-year OS as candidate factors for recursive-partitioning analysis (RPA). RPA was performed using the survival analysis trees method (https://ysph.yale.edu/c2s2/software/stree/) to divide the population into different risk subgroups [15]. A two-sided *P *value < 0.05 was considered statistically significant.

## Results

The mean age of the 116 patients was 57.3 ± 9.9 years-old; 103 patients (88.8%) were male. The demographic characteristics of the 116 patients are shown in Table 1. The p16-positive subgroup had a lower frequency of betel nut consumption (48.0%, *p* = 0.002), a lower frequency of cigarette smoking (44.0%, *p* = 0.007), a higher percentage of females (32.0%, *p* < 0.001), a higher frequency of high CPS for PD-L1 (56.0%, *p* = 0.046), and a higher frequency of tonsillar sub-sites (72.0%, *p* = 0.001), compared with the p16-negative group (Table 1).

**Table 1:** Demographic and clinical characteristics of study patients, n = 116

Variable	All (n = 116)	p16− (n = 91)	p16 + (n = 25)	*P* value
Age (Mean ± SD)	57.3 ± 9.9	57.2 ± 10.5	57.8 ± 7.1	0.744
Sex				< 0.001
Female	13 (11.2)	5 (5.5)	8 (32.0)	
Male	103 (88.8)	86 (94.5)	17 (68.0)	
Alcohol	84 (72.4)	69 (75.8)	15 (60.0)	0.117
Betel nuts	84 (72.4)	72 (79.1)	12 (48.0)	0.002
Cigarettes	77 (66.4)	66 (72.5)	11 (44.0)	0.007
High PD-L1 (CPS ≥ 20)	45 (38.8)	31 (34.1)	14 (56.0)	0.046
P53 mutant (≥ 80%)	22 (19.0)	20 (22.0)	2 (8.0)	0.114
High EGFR expression (H score ≥ 200)	34 (29.3)	30 (33)	4 (16)	0.099
Tumor subsite				0.004
Tonsil	47 (40.5)	29 (31.9)	18 (72.0)	
Soft palate	35 (30.2)	32 (35.2)	3 (12.0)	
Tongue base	27 (23.2)	23 (25.2)	4 (16.0)	
Others*	6 (5.1)	6(6.6)	0	
Differentiation				0.068
Well/moderately	86 (74.1)	71 (78.0)	15 (60.0)	
Poorly	30 (25.9)	20 (22.0)	10 (40.0)	
Treatment				0.008
Operation	59 (50.9)	53 (58.2)	6 (24.0)	
CCRT/RT	33 (28.4)	21 (23.1)	12 (48.0)	
IC-based treatment**	24 (20.7)	17 (18.7)	7 (28.0)	
cT classification				0.199
T1	42 (36.2)	33 (36.3)	9 (36.0)	
T2	38 (32.8)	28 (30.8)	10 (40.0)	
T3	11 (9.5)	7 (7.7)	4 (16.0)	
T4	25 (21.6)	23 (25.3)	2 (8.0)	
cN classification				0.086
N0	61 (52.6)	53 (58.2)	8 (32.0)	
N1	6 (5.2)	4 (4.4)	2 (8.0)	
N2	47 (40.5)	32 (35.2)	15 (60.0)	
N3	2 (1.7)	2 (2.2)	0 (0.0)	
cStage				0.255
I	30 (25.9)	25 (27.5)	5 (20.0)	
II	26 (22.4)	23 (25.3)	3 (12.0)	
III	8 (6.9)	5 (5.5)	3 (12.0)	
IV	52 (44.8)	38 (41.8)	14 (56.0)	
Extranodal extension***	32 (29.1)	22 (25.6)	10 (41.7)	0.125

*CCRT/RT* Concurrent chemo-radiotherapy/radiotherapy; *CPS* Combined positive score, *EGFR* Epidermal growth factor receptor, *ENE* Extra-nodal extension, *IC* Induction-based chemotherapy, *SD* Standard deviation, *PD-L1* Programmed cell death ligand 1

*Unclear tumor subsite or extension beyond the oropharynx

**7 patients with chemoradiotherapy; 7 patients with surgery; 7 patients with surgery and chemoradiotherapy

***Six patients had missing extra-nodal extension (ENE) data due to no available image study: one in p16 positive OPC and five in p16 negative OPC

**Fig. 1:**
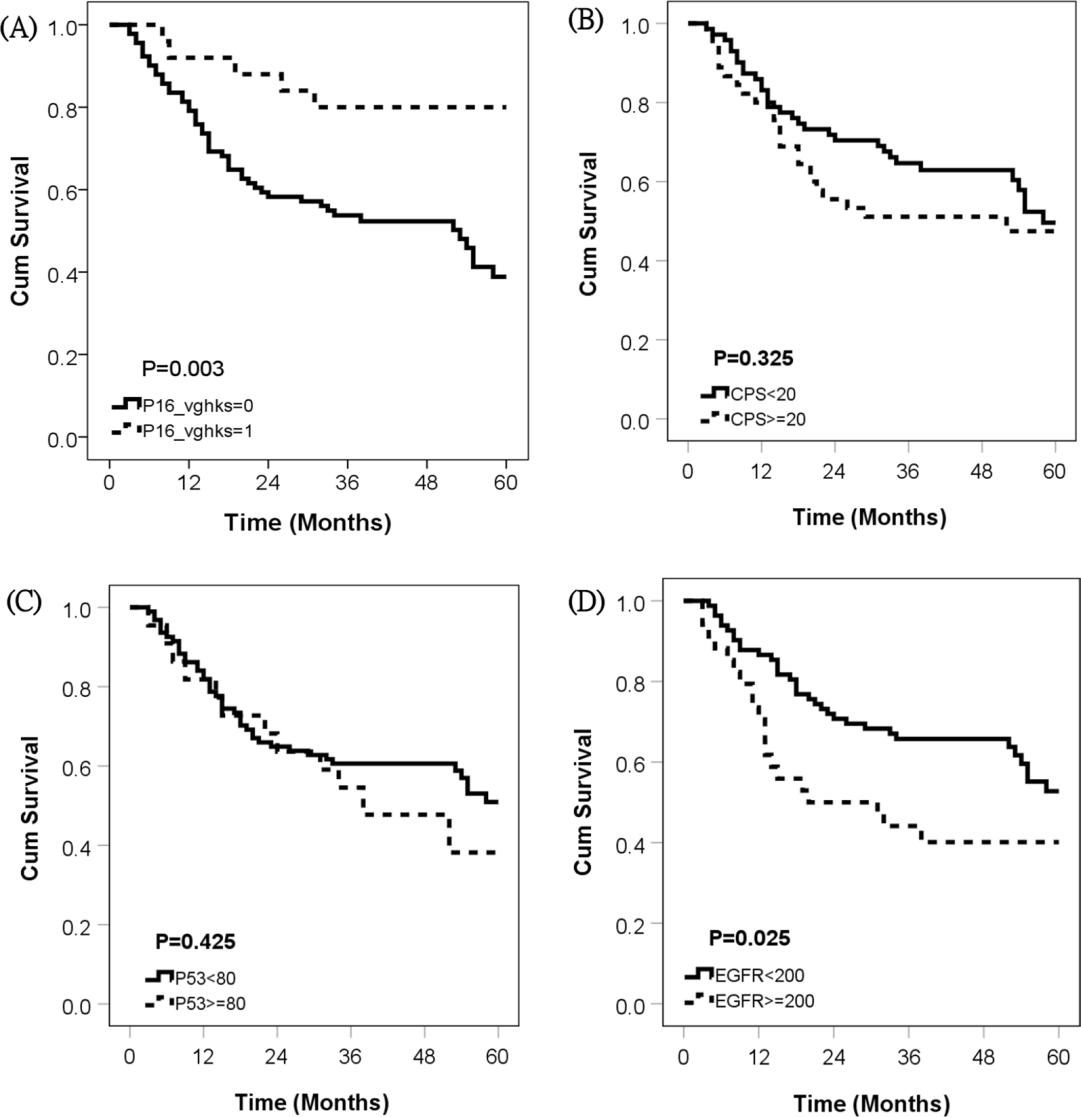
Kaplan Meier plot stratified cumulative survival by **A** p16 (*p* = 0.003), **B** PD-L1 CPS (*p* = 0.325), **C** p53 (*p* = 0.425), and **D** EGFR (*p* = 0.025), respectively. (*Abbreviations* CPS: combined positive score; EGFR: epidermal growth factor receptor; PD-L1: programmed cell death ligand 1)

In survival analysis of the tested biomarkers, p16-positive patients had a significantly lower risk of mortality (hazard ratio [HR] 0.274, *p* = 0.006) than p16-negative patients (Table 2 and Fig. 1). Patients with high EGFR expression (defined as a H score ≥ 200) had a significantly higher risk of mortality (HR 1.86, *p* = 0.028) compared to patients with low EGFR expression. PD-L1 and p53 status had no significant impact on mortality (*p* = 0.237 and *p* = 0.429, respectively).

**Table 2:** Impact of biomarkers on mortality

Variable	Total	Event (%)	Hazard ratio	*P* value
p16 status				0.006
p16 negative	91	49 (53.8)	Ref.	
p16 positive	25	5 (20.0)	0.274	
PD-L1				0.237
CPS < 20	71	31 (43.7)	Ref.	
CPS ≥ 20	45	23 (51.1)	1.386	
p53 mutant				0.429
No	94	42 (44.7)	Ref.	
Yes	22	12 (54.5)	1.296	
High EGFR expression				0.028
No (H-score < 200)	82	34 (41.5)	Ref.	
Yes (H-score ≥ 200)	34	20 (58.8)	1.86	

*CPS* Combined positive score, *EGFR* Epidermal growth factor receptor, *PD-L1* programmed cell death ligand 1

The variables that had a significant negative impact on five-year OS in Cox regression were age {HR 1.03 [95% confidence interval (CI) 1.001‒1.06]}, p16 negativity (HR 0.27 [95% CI 0.11‒0.69], *p* = 0.006), high EGFR expression (HR 1.86 [95% CI 1.07‒3.24]), cT4 (HR 3 [95% CI 1.7‒5.29]), extra-nodal tumor extension (HR 1.85 [95% CI 1.04‒3.28]), alcohol consumption (HR 2.17 [95% CI 1.06‒4.44]), and cigarette smoking (HR 2.06 [95% CI 1.09‒3.92]; all *p* < 0.05; Table 3).

**Table 3:** Hazard ratios for 5-year overall survival

Variable	HR (95% CI)	*P* value
Age	1.03 (1.001–1.06)	0.042
Male	2.75 (0.86–8.84)	0.089
P16 positive	0.27 (0.11–0.69)	0.006
PD-L1	1.31 (0.76–2.25)	0.329
P53 mutant	1.30 (0.68–2.45)	0.429
High EGFR expression	1.86 (1.07–3.24)	0.028
*Tumor subsite*		
Tonsil	Ref.	
Soft palate	1.36 (0.70–2.67)	0.367
Others	1.67 (0.87–3.20)	0.121
Differentiation-poorly	1.40 (0.78–2.51)	0.258
*Treatment*		
Operation	Ref.	
CCRT/RT	1.4 (0.77–2.54)	0.27
IC-based treatment	0.96 (0.45–2.04)	0.909
cT4	3 (1.7–5.29)	< 0.001
cN2-N3	1.50 (0.88–2.57)	0.137
cStage: III–IV	1.54 (0.90–2.64)	0.117
Extranodal extension	1.85 (1.04–3.28)	0.035
Alcohol	2.17 (1.06–4.44)	0.034
Betel nuts	1.40 (0.74–2.66)	0.304
Cigarettes	2.06 (1.09–3.92)	0.027

*CCRT/RT* Concurrent chemo-radiotherapy/radiotherapy, *95%CI* 95% confidence interval, *EGFR* Epidermal growth factor receptor, *HR* Hazard ratio, *IC* Induction-based chemotherapy, *PD-L1* Programmed cell death ligand 1

**Fig. 2:**
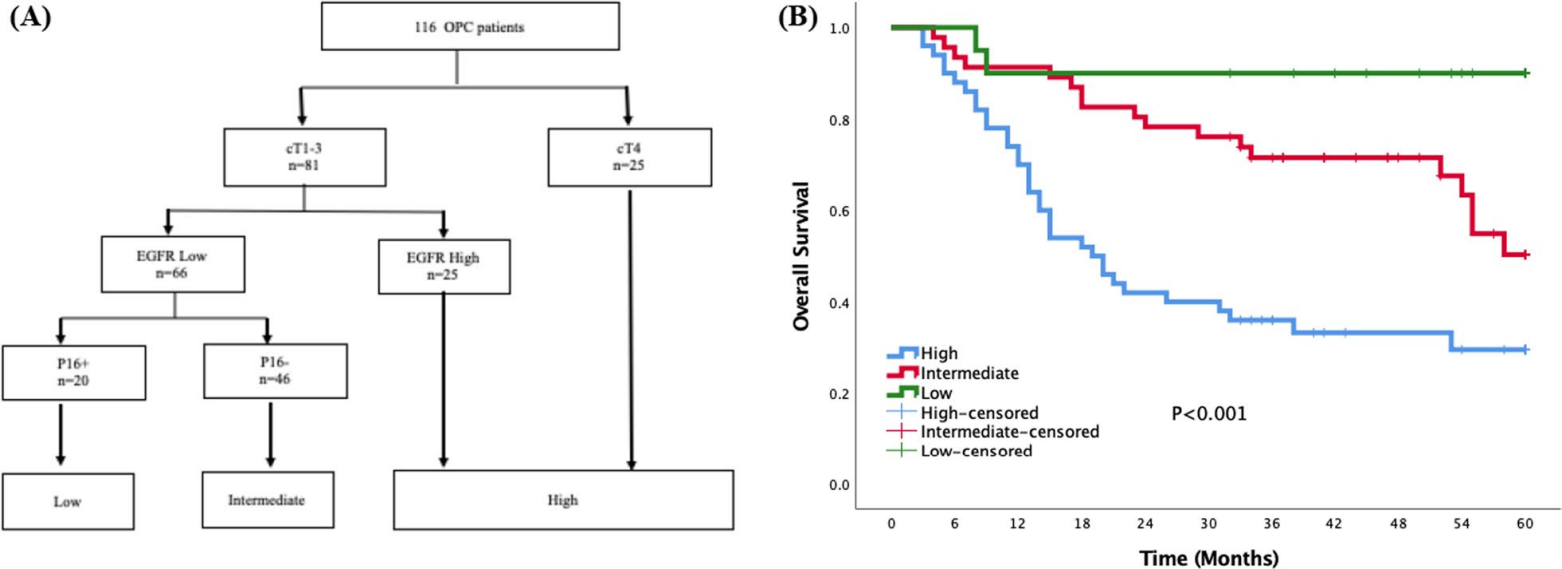
Classification of oropharyngeal cancer patients into three categories (**A**) and 5-year overall survival according to those categories (**B**)

The significant variables in univariate analysis were included in survival tree analysis. The 116 patients with OPC were classified into high-risk, intermediate risk, and low-risk categories using survival tree analysis. The foremost significant predictive parameter was cT stage; all patients with cT4 OPC were classified into the high-risk group. Patients with cT1 ~ 3 stage OPC and high EGFR expression were also classified into the high-risk group. Patients with cT1 ~ 3 stage OPC and low EGFR expression were further stratified according to p16 status: p16-positive OPC was classified into the low-risk group and p16-negative OPC was classified into the intermediate risk group (Fig. 2A). Five-year OS for the high-, intermediate, and low-risk groups was 29.5%, 50.3%, and 90%, respectively (*p* < 0.001; Fig. 2B).

## Discussion

The superior prognosis of HPV-related (p16-positive) OPC has been recognized by establishment of a distinct staging system for this subtype in the AJCC 8th edition [5]. P16 serves a surrogate marker of HPV relatedness and correlated well with HPV DNA in previous large-scale studies of OPC [1, 14]. However, relatively few studies have investigated risk stratification of patients with OPC from regions with a low incidence of HPV and where betel nut is commonly chewed. In Taiwan where this study was conducted, the prevalence of HPV in OPC has remained low [16]. Moreover, given that betel nut chewing is popular in this region, the survival trends for patients with OPC from Taiwan may not be identical to patients in HPV-prevalent areas.

In this study, we identified the variables that significantly influence survival among patients with OPC from Taiwan using Cox regression and then employed survival tree analysis to prioritize these parameters to generate a risk prediction model. We found that p16-positive patients had significantly better OS compared to p16-negative patients and patients with high EGFR expression had significantly worse OS compared to those with low EGFR expression, as evidenced by Kaplan–Meier survival curves. The cT stage was the foremost parameter in the risk stratification model developed using survival tree analysis, followed by EGFR expression, and then p16 status. The 5-year OS rates for the high-, intermediate, and low-risk groups were 29.5%, 50.3%, and 90%. Thus, the risk stratification model developed in this study could provide useful information for physicians in non-western countries.

High EGFR expression is associated with a poor prognosis in OPC [17], including a subset of HPV-related OPC [6]. Various mechanisms have been proposed to explain the association between EGFR over-expression and poorer prognosis [18, 19]. Cigarette smoking is carcinogenic and is related to high EGFR expression, possibly through induction of local hypoxia [6, 19]. Moreover, EGFR plays a role in endoplasmic reticulum stress signaling that leads to radio-resistance in OPC [18]. Combined evaluation of p16 and EGFR in previous large-scale studies of OPC found that p16-positive/low EGFR was associated with the best survival outcomes whereas p16-negative/high EGFR OPC had the poorest survival outcomes [6, 19]. Similarly, our survival tree analysis categorized early-stage p16-positive/low EGFR OPC into the low-risk group.

Recursive-partitioning analysis can readily separate patients with OPC from countries where HPV is prevalent into two comparably- sized groups based on HPV relatedness, with HPV as the most influential prognostic predictor [14]. However, the same conclusion cannot be reached worldwide, as the prevalence of HPV is low in some regions, such as Taiwan. Tumor stage is the most important factor in survival trees for OPC in areas where HPV is prevalent [14], and advanced cT stage was identified as the most important prognostic factor in our study. Although EGFR expression was not included in a previously described survival tree [14], EGFR was suggested as a biomarker of smoking status in another study [19]. EGFR expression appeared to be the second most influential prognostic factor in our study, which further implies the importance of cigarette smoking in OPC risk stratification suggested in the previous literature [14].

In our study, p16-positive OPC was associated with a significantly higher percentage of high CPS for PD-L1, which is in concordance with a previous study that suggested PD-L1 is more frequently expressed in HPV-related OPC than HPV-negative OPC [9]. Higher PD-L1 CPS has been suggested to be associated with a better response to PD-1 pathway inhibition therapy compared to tumors with low PD-L1 CPS in a pre-treated group of patients with head and neck cancer [8]. However, OPC with higher PD-L1 expression had no significant prognostic advantage over OPC with lower PD-L1 when receiving primary radiotherapy alone [20]. Although one meta-analysis found higher PD-L1 was associated with better prognosis in OPC, treatment modality was unable to be considered individually [21]. In another study, the level of PD-L1 expression demonstrated no significant association with overall survival in oropharyngeal and oral cancer [22]. Therefore, the apparent survival benefit of PD-L1 expression may largely be related to the response to PD-1 pathway inhibition therapy [8]. In our research, the patients did not receive immunotherapy in the study interval, and we found no significant association between the PD-L1 CPS and OS in OPC.

There are several limitations to this work. This was a single-institute, retrospective study of a relatively limited number of cases. Exclusion of 23 OPC patients due to missing data can introduce potential selection bias. The exact cigarette smoking status was not recorded in pack-years, which made it difficult to stratify the cumulative risk associated with cigarette smoking. Although in situ hybridization to detect HPV DNA was not available in this study, p16 immunohistochemistry correlates well with HPV DNA and can serve as a surrogate marker for HPV [1, 4, 14, 23]. P16 immunohistochemistry status has also been adopted by the AJCC 8th edition as a proxy for HPV-relatedness in OPC staging [5]. However, p16 immunohistochemistry still lacks specificity for transcriptionally active HPV even though it has been shown to be a good surrogate marker for HPV positivity. The AJCC 8th edition eliminated ENE in nodal staging of p16-positive OPC [5]. However, a recent large-scale study indicates the importance of ENE cannot be overlooked in HPV-related OPC [24]. Therefore, we restaged our OPC cohort using the AJCC 7th edition [25] in order to assess the influence of ENE on OS, regardless of HPV-relatedness. Among the patients treated with IC, the choice of subsequent radiotherapy/chemoradiotherapy and surgery was not clearly recorded. It prevented us from further exploring the impact of IC on survival rates. Future studies are warranted to further elucidate risk stratification and identify more precise treatment strategies for different subgroups of patients with OPC.

## Conclusion

Owing to the low prevalence of HPV and popularity of betel nut chewing, the relative importance of prognostic factors in OPC is not identical in Asian and Western countries. Survival tree analysis indicates the most influential parameter for risk stratification for patients with OPC from Taiwan is cT stage, followed by EGFR expression, and then p16 status. Further analysis of significant prognostic biomarkers in regions with a low prevalence of HPV and where betel nut chewing is popular may facilitate the design of improved of treatment strategies for OPC in the future.

## Supplementary Information

**Additional file 1: Figure S1A.** P16 positivity was defined as diffuse, strong nuclear and cytoplasmic staining in ≥ 70% tumor cells.

**Additional file 2: Figure S1B.** P16 negative. (Scale bar= 100 µm).

**Additional file 3: Figure S2A**. Strong nuclear p53 staining in ≥ 80% tumor cells was recorded as p53 mutant-type.

**Additional file 4: Figure S2B**. Tumor cells with almost no P53 immunostaining. (Scale bar= 100 µm).

**Additional file 5: Figure S3A.** High EGFR expression.

**Additional file 6: Figure S3B.** Low EGFR expression. (Scale bar= 100 µm).

**Additional file 7: Figure S4A**. High PD-L1 expression.

**Additional file 8: Figure S4B**. Low PD-L1 expression. (Scale bar=100 µm).

## Data Availability

The data that support the findings of this study are available on request from the corresponding author. The data are not publicly available due to privacy or ethical restrictions.

## Abbreviations

AJCC: American Joint Committee on Cancer
CI: Confidence interval
CPS: Combined positive score
EGFR: Epidermal growth factor receptor
ENE: Extra-nodal tumor extension
HPV: Human papillomavirus
HR: Hazard ratio
IHC: Immunohistochemical
IRB: Institutional Review Board
OPC: Oropharyngeal cancer
OS: Overall survival
PD-1: Programmed death 1
PD-L1: Programmed cell death ligand 1
RPA: Recursive-partitioning analysis
TMA: Tissue microarray
