# Supplementary figures and images for: Prognostic stratification of oropharyngeal cancer patients in a betel nut chewing and low HPV area
Source: J Otolaryngol Head Neck Surg. 2023 Apr 20;52:27. doi: 10.1186/s40463-023-00632-x (PMC10116661; doi:10.1186/s40463-023-00632-x)

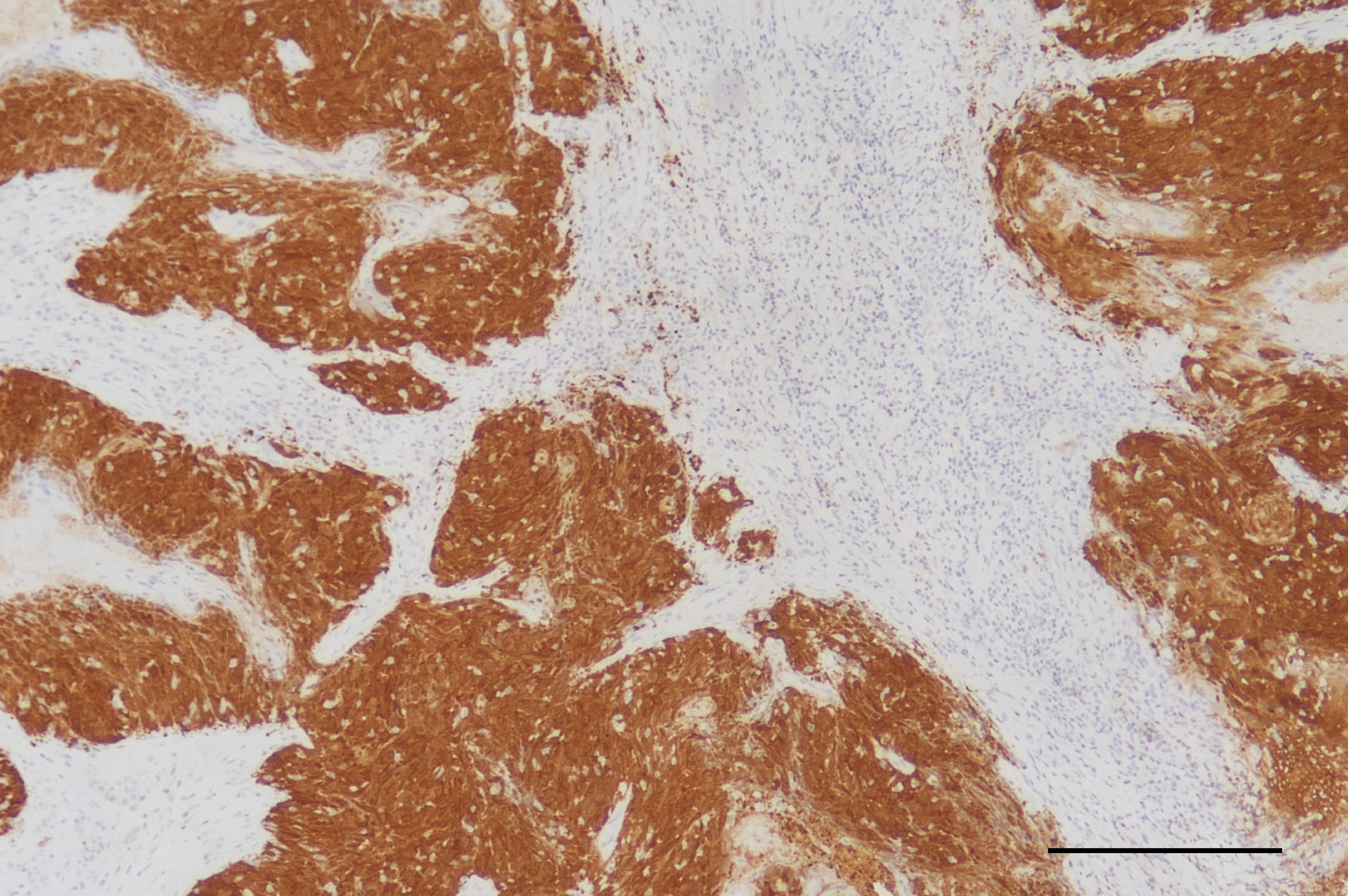

Supplement: Supplementary file 1 — Additional file 1: Figure S1A. P16 positivity was defined as diffuse, strong nuclear and cytoplasmic staining in ≥ 70% tumor cells. [file 40463_2023_632_MOESM1_ESM.tif]

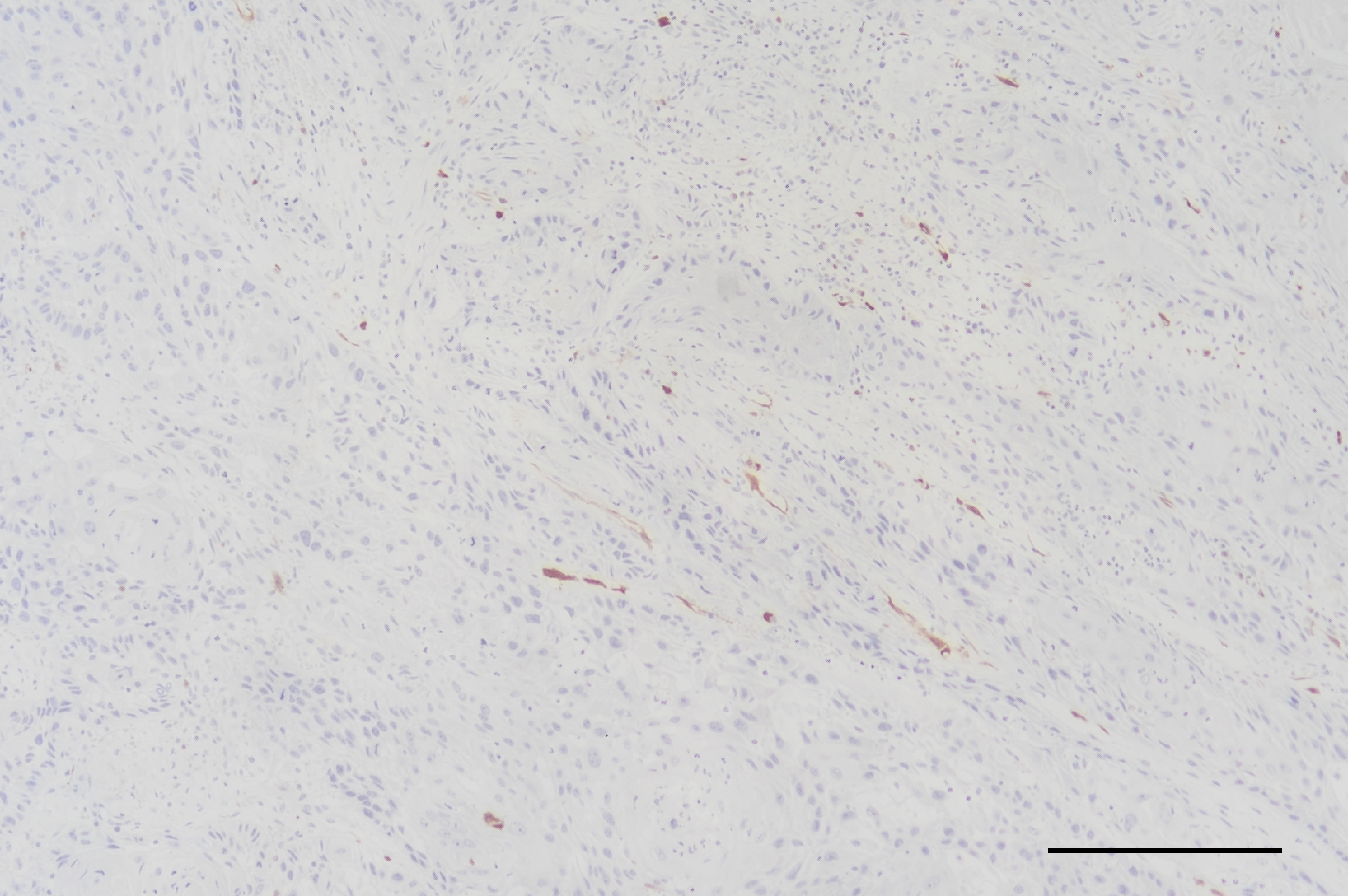

Supplement: Supplementary file 2 — Additional file 2: Figure S1B. P16 negative. (Scale bar= 100 µm). [file 40463_2023_632_MOESM2_ESM.tif]

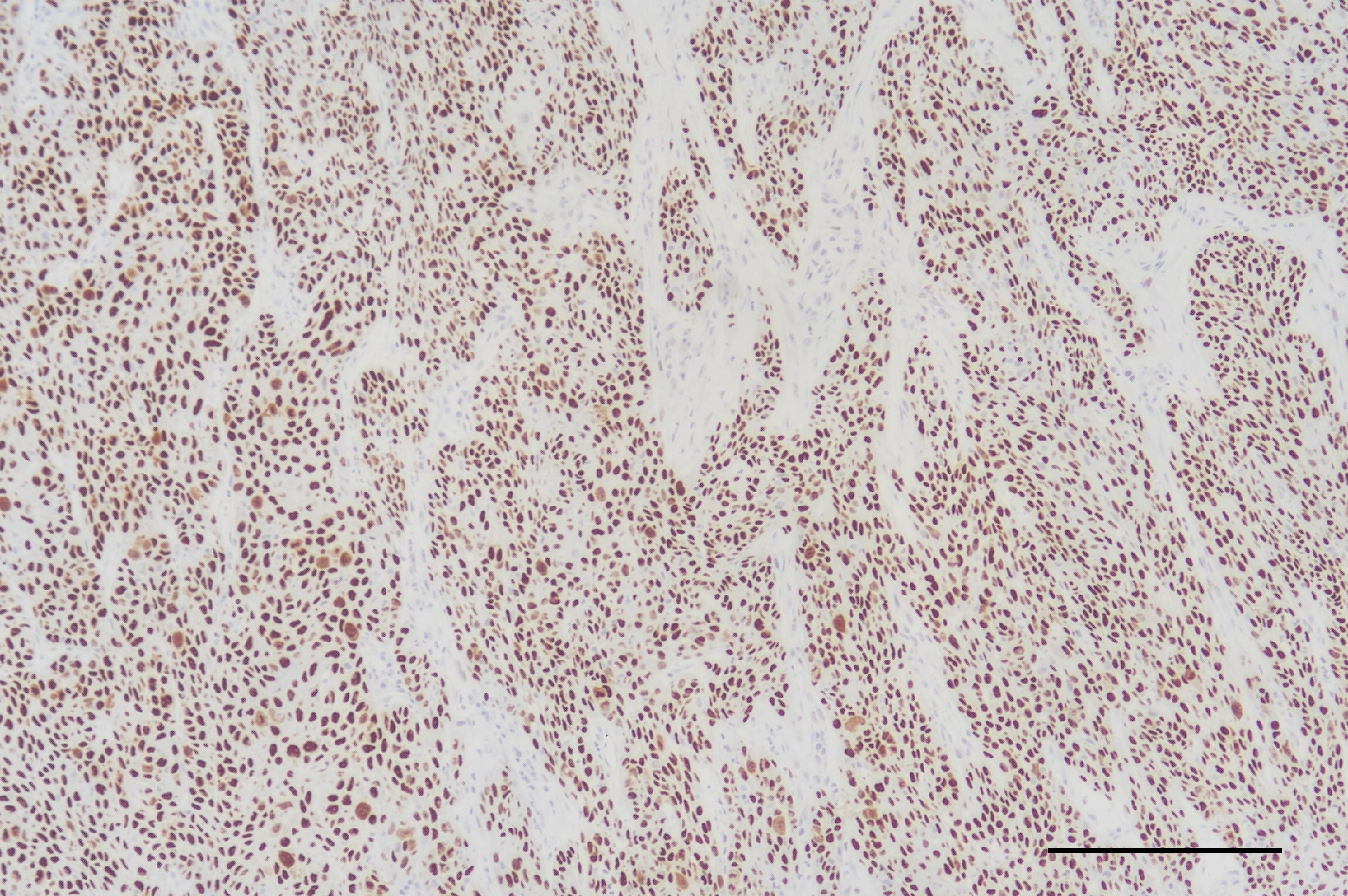

Supplement: Supplementary file 3 — Additional file 3: Figure S2A. Strong nuclear p53 staining in ≥ 80% tumor cells was recorded as p53 mutant-type. [file 40463_2023_632_MOESM3_ESM.tif]

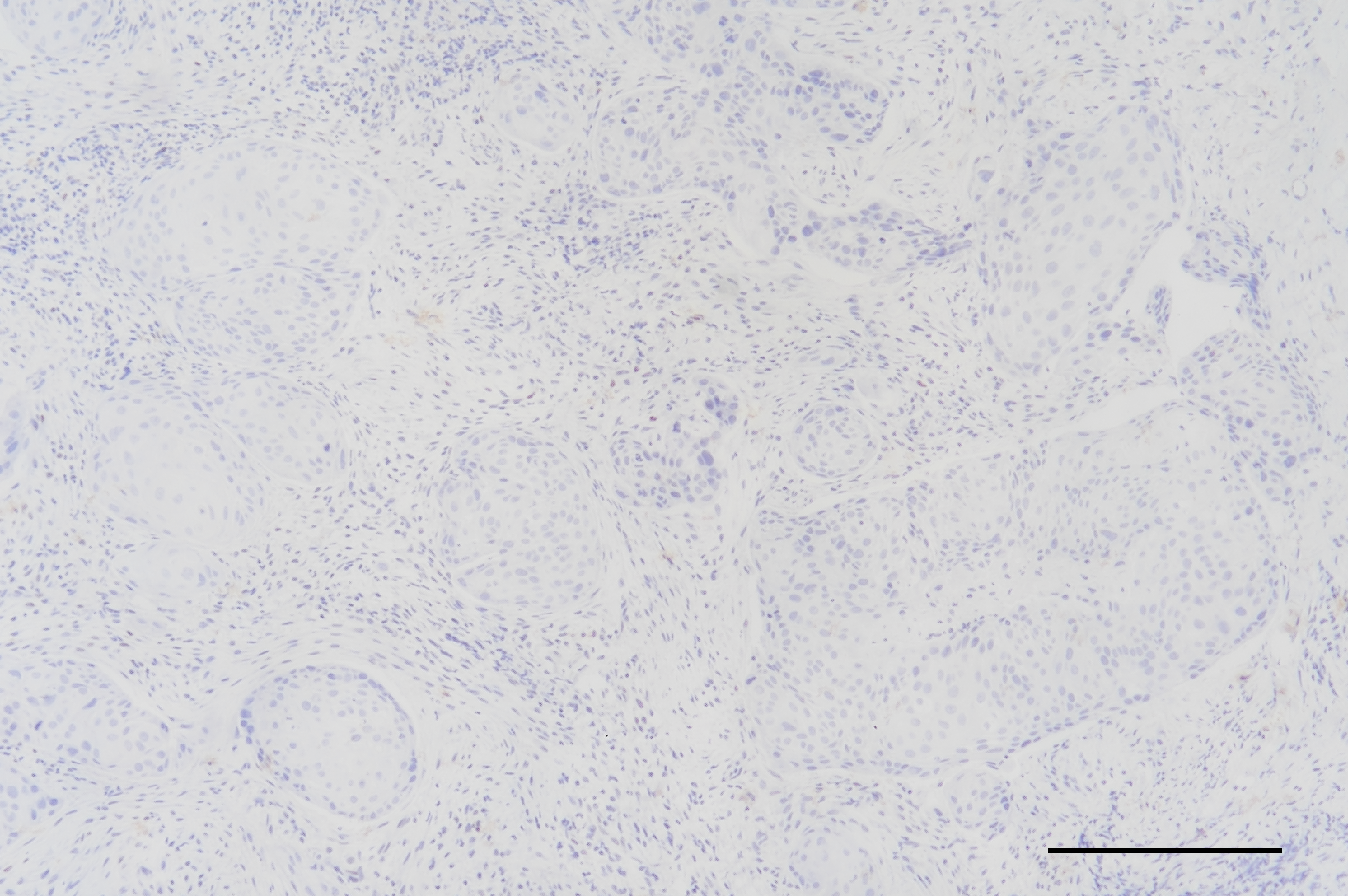

Supplement: Supplementary file 4 — Additional file 4: Figure S2B. Tumor cells with almost no P53 immunostaining. (Scale bar= 100 µm). [file 40463_2023_632_MOESM4_ESM.tif]

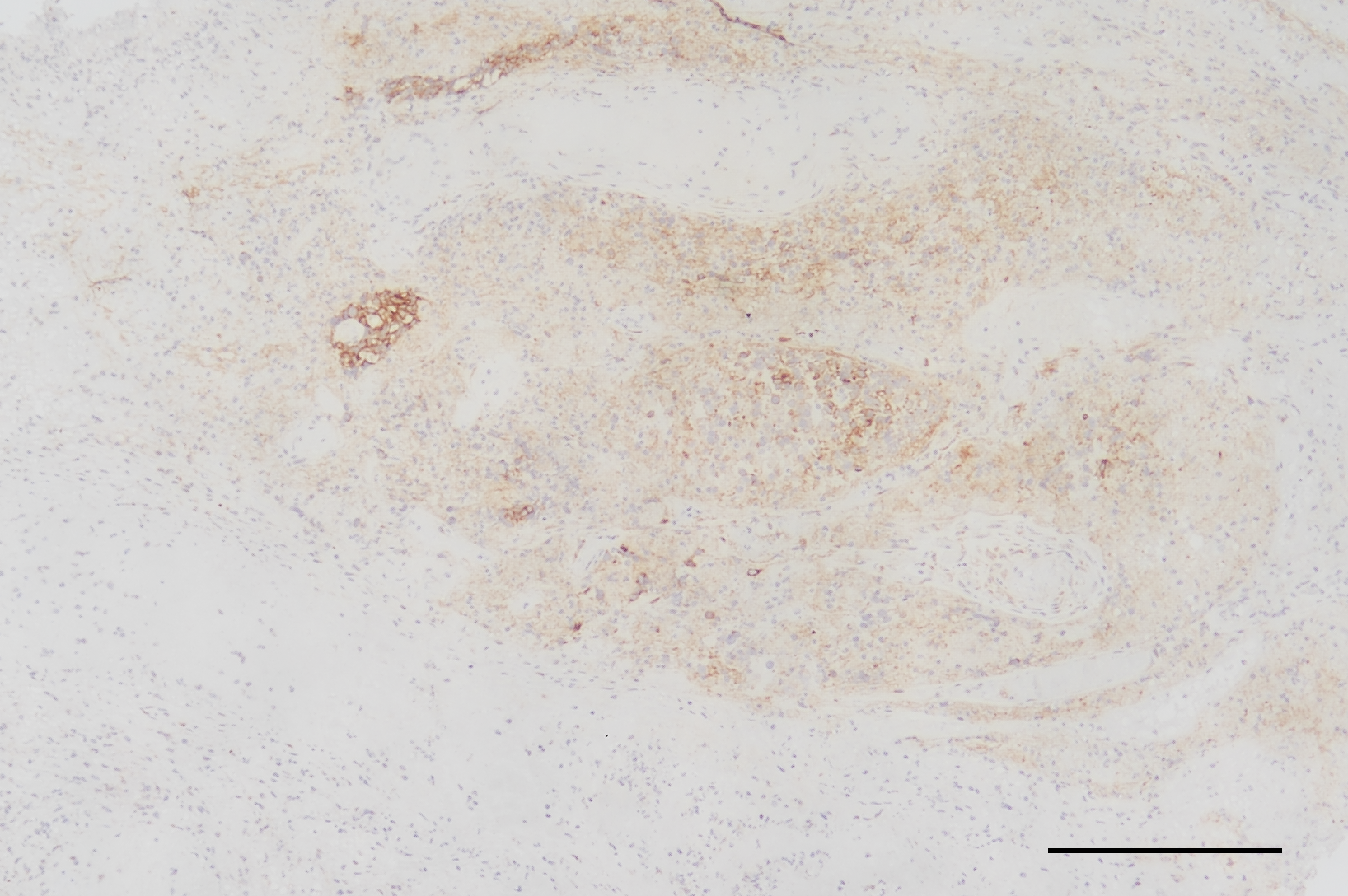

Supplement: Supplementary file 6 — Additional file 6: Figure S3B. Low EGFR expression. (Scale bar= 100 µm). [file 40463_2023_632_MOESM6_ESM.tif]

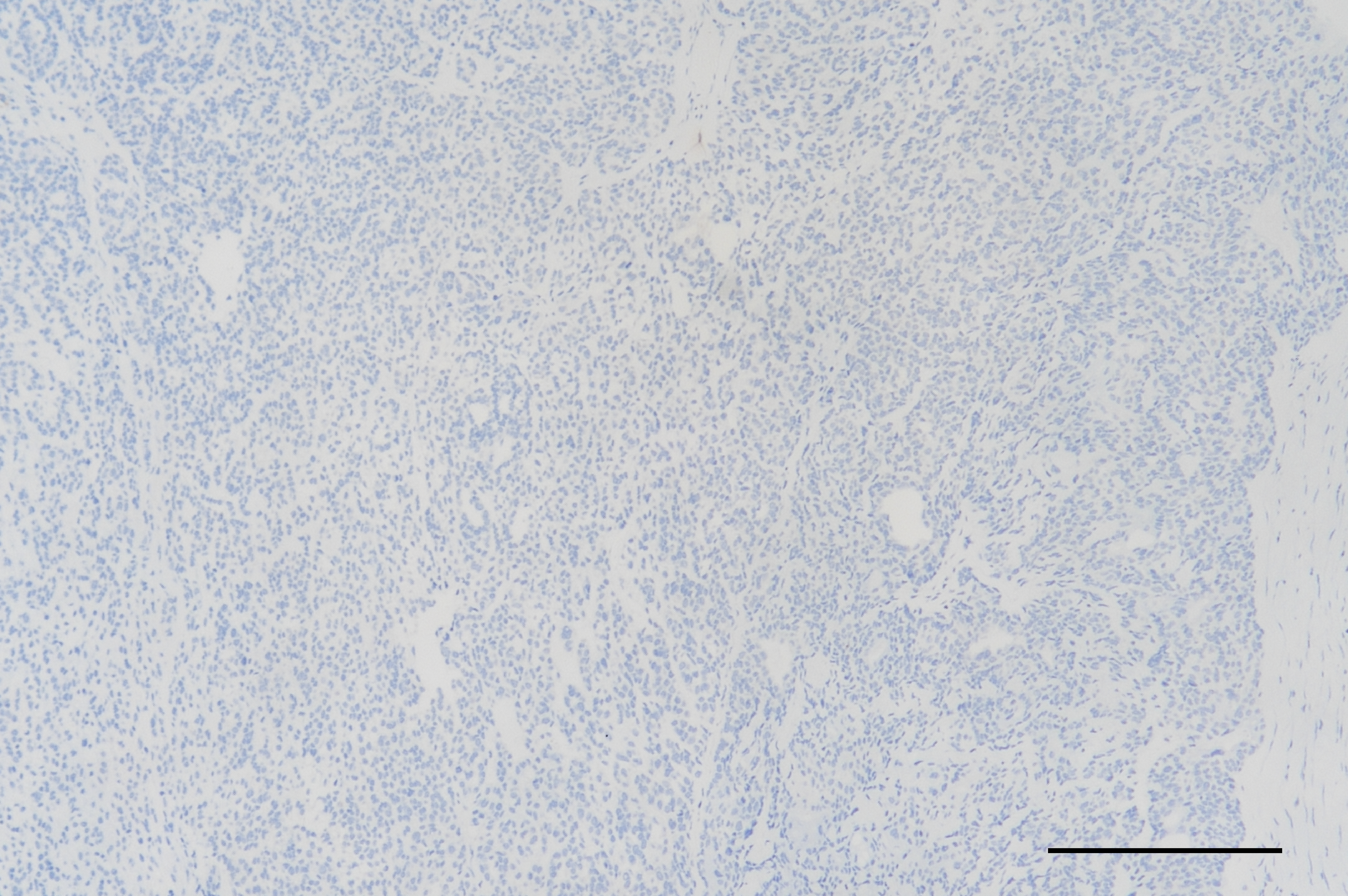

Supplement: Supplementary file 8 — Additional file 8: Figure S4B. Low PD-L1 expression. (Scale bar=100 µm). [file 40463_2023_632_MOESM8_ESM.tif]
